# Massage Therapy’s Effectiveness on the Decoding EEG Rhythms of Left/Right Motor Imagery and Motion Execution in Patients With Skeletal Muscle Pain

**DOI:** 10.1109/JTEHM.2021.3056911

**Published:** 2021-02-03

**Authors:** Huihui Li, Kai Fan, Junsong Ma, Bo Wang, Xiaohao Qiao, Yan Yan, Wenjing Du, Lei Wang

**Affiliations:** 1 Shenzhen Institute of Advanced TechnologyChinese Academy of SciencesShenzhen518055China; 2 North China Institute of Aerospace Engineering 117817 Langfang065000China; 3 School of Electronic Engineering and AutomationGuilin University of Electronic Technology 71207 Guilin541004China; 4 Electronic and Communication Engineering DepartmentWuhan University of Technology 12565 Wuhan430070China

**Keywords:** Motor imagery (MI), motion execution (ME), electroencephalograph (EEG), classification, convolutional neural networks (CNN), attention-based bi-directional long short-term memory (BiLSTM), permutation disalignment index (PDI), artifact removal

## Abstract

Objective: Most of effectiveness assessments of the widely-used Massage therapy were based on subjective routine clinical assessment tools, such as Visual Analogue Scale (VAS) score. However, few studies demonstrated the impact of massage on the Electroencephalograph (EEG) rhythm decoding of Motor imagery (MI) and motion execution (ME) with trunk left/right bending in patients with skeletal muscle pain. Method: We used the sample entropy (SampEn), permutation entropy (PermuEn), common spatial pattern (CSP) features, support vector machine (SVM) and logic regression (LR) classifiers. We also used the convolutional neural network (CNN) and attention-based bi-directional long short-term memory (BiLSTM) for classification. Results: The averaged SampEn and PermuEn values of alpha rhythm decreased in almost fourteen channels for five statuses (quiet, MI with left/right bending, ME with left/right bending). It indicated that massage alleviates the pain for the patients of skeletal pain. Furthermore, compared with the SVM and LR classifiers, the BiLSTM method achieved a better area under curve (AUC) of 0.89 for the classification of MI with trunk left/right bending before massage. The AUC became smaller after massage than that before massage for the classification of MI with trunk left/right bending using CNN and BiLSTM methods. The Permutation direct indicator (PDI) score showed the significant difference for patients in different statuses (before vs after massage, and MI vs ME). Conclusions: Massage not only affects the quiet status, but also affects the MI and ME. Clinical Impact: Massage therapy may affect a bit on the accuracy of MI with trunk left/right bending and it change the topography of MI and ME with trunk left/right bending for the patients with skeletal muscle pain.

## I. Introduction

Massage is a widely-used complementary and alternative therapy in treating patient with skeletal muscle pain. Most of the previous effectiveness assessment were based on subjective routine clinical assessment tools [1], such as 10-point Visual Analogue Scale (VAS) score [2], [3], the short form McGill Pain Questionnaire (MPQ) [2], state anxiety inventory (STAI) [3], [4], Hospital Anxiety and Depression Scale (HADS), Oswestry Disability Index (ODI) [5] for disability measurement, and hospital stay [6]. Although these tools have clinical meaning, they are subjective and rough. Electroencephalogram (EEG) is non-invasive, cheap and convenient. In the existing studies using EEG to evaluate the effect of MT [7]–[9], most analysis focus on EEG absolute spectral power of four rhythms, such as delta (0–4 Hz), theta (4–8 Hz), alpha (mu, 8–13 Hz) and beta (13–30 Hz) activity [10]. In our previous work, we have used entropy-based method for the EEG evaluation during the quiet status [11].

Motor imagery (MI) is described as the concept of imagining a motor task without resulting in physical execution [12], [13]. As an important paradigm of spontaneous brain computer interfaces (BCI), MI has been widely used in the rehabilitation for the motor dysfunction patients [14]–[17] (facial palsy [15] and knee arthroplasty [17]), disorders of consciousness, attention deficit hyperactivity disorder, schizophrenia, epilepsy, and autism spectrum disorder [18]. BCI translates EEG signals into control commands so that physically impaired patients can control assistive devices. Therefore, the classification of MI is an important issue and hot topic in recent years [19]–[22].

Motor Execution (ME) is the actual practice of the movement, which is opposed to the MI [12]. Previous studies showed that MI and ME for hand and foot movements activate comparable brain areas, also for the execution of swallowing [23]. MI movement pattern discrimination were based on quantification of event-related synchronization/desynchronization (ERS/ERD) using bandpower (BP) [12], [24], which were cortical rhythms characterized by the mu and beta neural activity patterns [25]. ERD showed stronger contra-lateralization features with movement intention and execution in the sensorimotor cortices, while ERS was found prominently in the ipsilateral hemisphere [25].

Very few studies analyze connectivity patterns revealed by EEG during MI and ME. Some of them focused on differences in connectivity patterns between MI and ME [12], [26]. The experimental method most frequently used to test the common-basis hypothesis for real vs. virtual movements is function magnetic resonance imaging (fMRI) [22], [27], Magnetoencephalography (MEG) [28], near infrared spectroscopy (NIRS) [29], and EEG. The primary motor cortex (M1) signals’ importance has been reported in providing the information necessary for BCI. NIRS detects the mean oxyhemoglobin (oxyHb) to reflect cerebral activation. However, the high cost of NIRS systems makes them less suitable for nonclinical settings [30]. Among all neuroimaging techniques, BCIs based on EEG are well accepted for practical applications because they are inexpensive, lightweight, portable, noninvasive with minimal clinical risks, user friendly, and comparatively easy to apply [24]. A previous study using a simple finger-tapping task suggested that the increasing of oxyHb levels in the supplemental motor area (SMA) and premotor area (PMA) during MI were similar to those observed during ME [31]. Chaisaen *et al.* [25] presented the decoding EEG rhythms during action observation, MI, and ME for the actions of standing and sitting.

However, most of these reports used simple movements as tasks (left/right hand, foot, and tongue). Furthermore, few studies reveal the impact of massage therapy on the EEG rhythm decoding of MI and ME among patients with skeletal pain. It is unclear whether massage affects the physiological brain signals (EEG) during the MI or the real motions. The combination of massage and MI/ME may lead to a new way to investigate pain relief of the brain. Thus, in this article, we focus on the massage effectiveness for the MI and ME with left/right bending. We selected trunk with left/right bending as the MI task because it is frequently used for patient with chronic skeletal pain undergoing rehabilitation.

The main contribution of the current study includes three parts. Firstly, the current study aimed to explore the difference of rhythms between MI and ME, showing the different cortical activation patterns in different statuses (before or after massage). Secondly, we compared the EEG entropy-based feature for the MI left/right motion and the corresponding ME. Combined with the different entropy-based features, SVM and LR classifiers, as well as convolutional neural network (CNN), were used to distinguish the resting versus task performance (the MI or ME). Thirdly, we quantified the effectiveness of massage for the classification of MI and ME of left/right bending in different statuses (before or after massage).

## II. Methods and Procedures

### A. Subjects

There are 71 participants volunteered to join this experiment. The demographic characteristics of patients is shown in Table 1. All data were expressed in mean values (mean) and standard deviation (std). All participants had no history of neurological or psychiatric disorders. They had Skeletal Muscle Pain (low back pain, neck pain or leg pain). The study was approved by the institutional review board of the Shenzhen Institutes of Advanced Technology, Chinese Academy of Sciences (SIAT-IRB-170815-H0171) and procedures were in accordance with the latest revision of the declaration of Helsinki. All subjects provided written informed consent.

**TABLE 1:** Demographic Characteristics of Participants (Mean ± Std)

Characteristics	Female (n=43)	Male (n=28)
Percentage (%)	60.5	39.4
Age (years)	53.39 (11.08)	55.71(10.20)
Height (cm)	161.63(4.91)	171.46(6.10)
Weight (kg)	60.72(6.23)	74.51(7.89)
Pain duration	≥6 months	≥6 months

### B. Experiment Procedure and Data Acquisition

**FIGURE 1.**
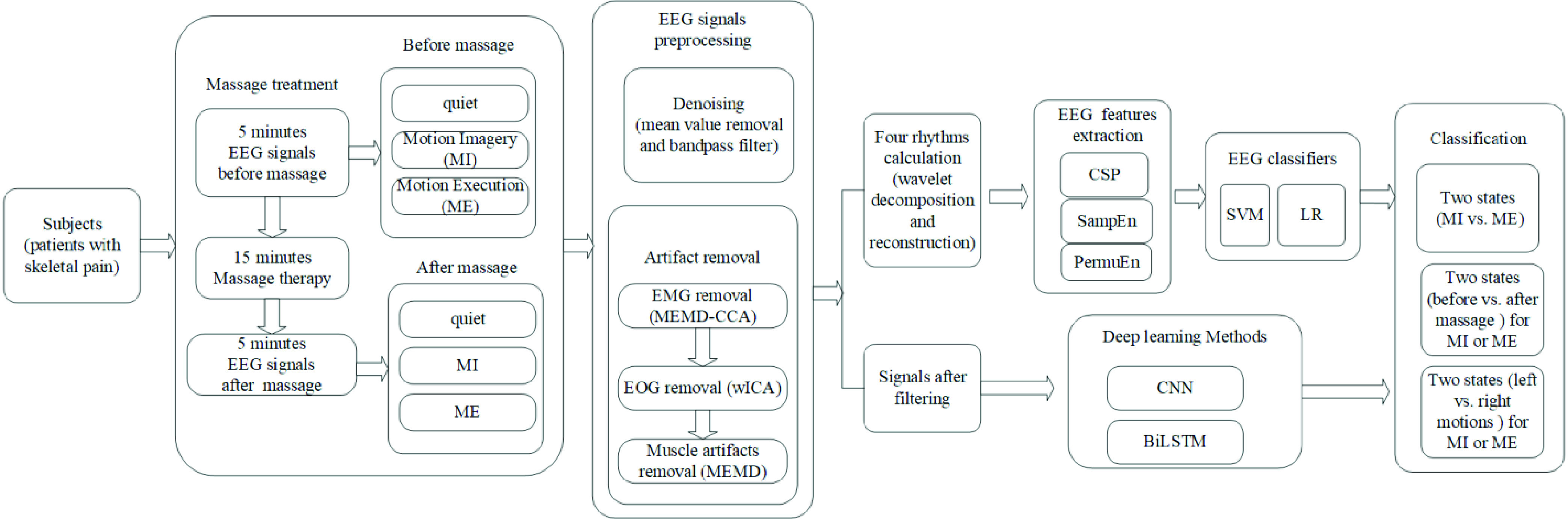
The total experiment procedure.

**FIGURE 2.**
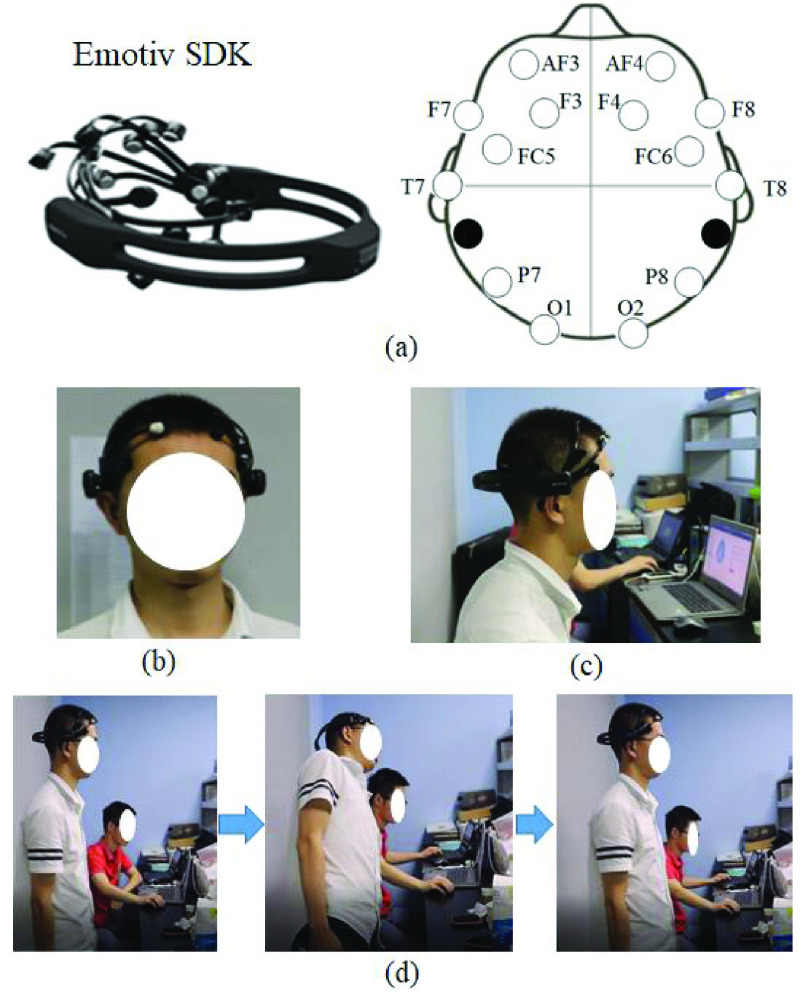
(a) Emotiv SDK equipment. (b) The front side of participant wearing Emotiv equipment. (c) Subject did the quiet test and MI with left/right bending while seating. (d) Subject did the real left bending.

The total experiment procedure was shown in Fig. 1. The Emotiv EPOC+ headset was used for the EEG data acquisition. It consists of 14 data channels (AF3, F7, F3, FC5, T7, P7, O1, O2, P8, T8, FC6, F4, F8, and AF4) and other two channels for references (Fig.2 (a)). The band-pass filter is set between 0.5 to 40 Hz, and sampling frequency is 128 Hz.

The experiment procedure was as follows. Before the patient received the massage therapy, patients were asked to close their eyes, sit alone on a chair quietly wearing the EEG headset and refrain from talking, falling asleep, or moving during the EEG measurement (Fig.2 (b)-(c)). It lasted 30 seconds. Then they were asked to close their eyes and image the motion with trunk left bending or right bending. The next task was to do the real motion with trunk left bending or trunk right bending. They were asked to stand straight first with eyes open, then they tried to move their trunk left or right, keeping the head not moving too heavily (Fig.2 (d)). At the same time, they listened to a recorder which was tapping five cycles of motions rhythms and followed its instruction. From the beginning to the end of the recorder, it lasted about 20 seconds with five cycle motions. The whole test task lasted about 20 minutes. The Chinese massage treatments were performed by professional massage therapists, and it was about half an hour for one subject.

After massage therapy, the patients did the EEG test with the repeated cycle.

### C. EEG Preprocessing Methods

In the preprocessing stage, artifact removal (electromyogram (EMG) and electrooculogram (EOG)) is a key to the EEG applications [12], [24]. EMG artifacts are caused by the electrical activities on the head surface from muscle movements and contraction and EMG activity has a broad frequency range, overlapping all classic EEG rhythms. Therefore, for experiments involving manipulations such as movement, it is quite difficult to avoid EMG artifacts.

**FIGURE 3.**
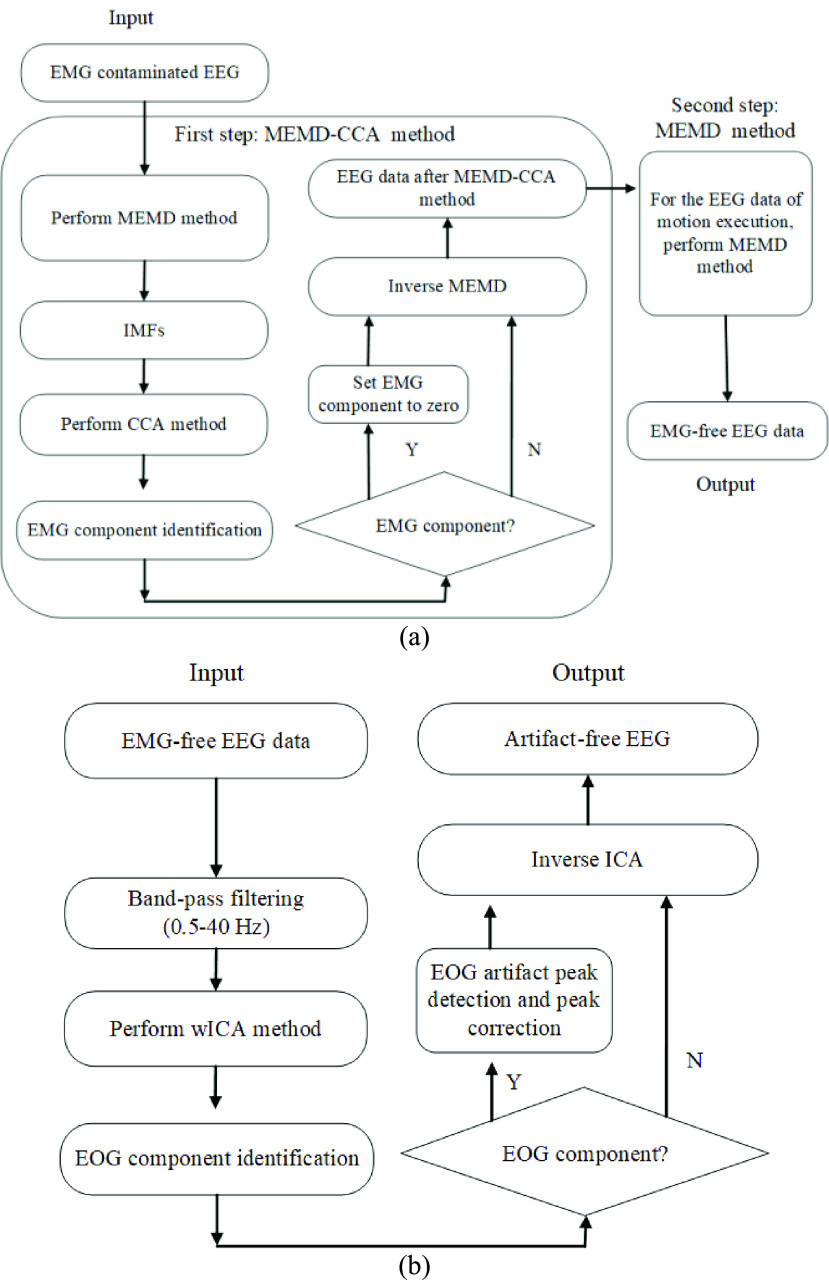
(a) Flowchart of the EMG artifact removal in the EEG signal. (b) Flowchart of the EOG removal in the EEG signal.

At present, the blind source separation algorithm (BSS) is used to separate the EEG and EMG sources into different components, and then remove the muscle-related components during the reconstruction process. BSS techniques mainly include independent component analysis (ICA) [32], [33], canonical correlation analysis (CCA) [34] and independent vector analysis (IVA) [35]. One fundamental requirement of the above multichannel techniques is that the number of channels must be larger than or equal to the number of underlying sources. To solve this issue, a number of algorithms have been proposed to decompose ambulatory EEG into multiple components, such as the wavelet transform, empirical mode decomposition (EMD) [36], ensemble empirical mode decomposition (EEMD) [37], multivariate empirical mode decomposition (MEMD) [38] and singular spectrum decomposition (SSA) [39]. Furthermore, the implementation algorithms of two-step strategy had been presented, such as wavelet ICA (wICA) [40], EEMD-ICA [41], EEMD-CCA [42], EEMD-IVA [43], MEMD-CCA [44], MEMD-IVA [45], and SSA-ICA [46] methods. Among these method, MEMD-CCA combined by MEMD and CCA has shown a good performance for muscle artifact removal in the few-channel setting [44]. MEMD-CCA retains EEG content in almost all channels, in contrast to EEMD-CCA. The CCA method outperformed a low-pass filter with different cutoff frequencies and an ICA-based technique for muscle artifact removal in a real ictal EEG recording [34], and it is more computationally efficient compared with ICA [30]. MEMD-CCA utilized inter-channel dependence information seen in the few-channel situation. Thus, we removed EMG artifact by MEMD-CCA (Fig. 3 (a)). Then we used the improved method based on wICA to remove EOG artifact (Fig.3(b)).

#### 1) EMG Artifact Removal

Firstly, MEMD was utilized to decompose 14-channel original EEG signals into multivariate intrinsic mode functions (IMFs) [38]. Then, CCA was applied to decompose the reorganized multivariate IMFs into the underlying sources S. Due to the broad frequency spectrum of EMG contamination in EEG recordings, muscle artifacts yield more properties of temporally white noise, thus they have a low autocorrelation [34]. The last several sources sorted by autocorrelation were assumed to correspond to muscle artifacts [44]. In our experiment, autocorrelated CCA sources with autocorrelation less than 0.3 were assumed to the muscle artifacts. By setting artifact-related sources to zero during reconstruction, other IMFs can be summed into the denoised EEG signal in each channel.

For the EEG data of ME, there were still obvious motion artifacts after we did the first step using MEMD-CCA method. Therefore, MEMD was used in the second step to decompose 14-channel reconstructed EEG data after MEMD-CCA method. It was observed that the EMG artifacts mainly existed in 6th-9th order IMFs, so 6th–9th order IMFs were set to zero and the other IMFs were reconstructed to obtain the artifact-free EEG data.

#### 2) EOG Artifact Removal

The improved wICA method has been presented to improve EOG artifact removal method [47] and outperformed other component rejection and wavelet-based EOG removal methods. The approach is to correct artifacts within the independent components instead of rejecting the entire component. This method preserves as much neural information as possible from the original signal [47].

In our experiment, after muscle artifacts removal, EMG-free EEG signals were 0.5–40 Hz band-pass filtered. Then, the filtered EEG data of 14 channels were decomposed by ICA method using EEGLAB’s runica function, and 14 independent component signals were obtained. If the independent components containing EOG artifacts [48] were identified by EEGLAB, a target window size of 1 second duration around the EOG artifact was used, as this spanned the length of the EOG artifact waveforms. We used the wavelet decomposition to remove EOG artifact in target window. Then, these components were used in the inverse ICA to reconstruct the cleaned EEG signal. The EEG preprocessing methods were implemented on Matlab (R2014b, MathWorks, United States).

After preprocessing, all data were segmented into each epoch with trial durations 4 s (512 points). The 10 different sub-sets include quiet without MI before massage (sub-set 1, 1724 epoches), quiet without MI after massage (sub-set 2, 1635 epoches), MI with left bending before massage (sub-set 3, 995 epoches), MI with left bending after massage (sub-set 4, 934 epoches), MI with right bending before massage (sub-set 5, 1038 epoches), MI with right bending after massage (sub-set 6, 975 epoches), real left bending before massage (sub-set 7, 1026 epoches), real left bending after massage (sub-set 8, 810 epoches), real right bending before massage (sub-set 9, 963 epoches), and real right bending after massage (sub-set 10, 865 epoches).

### D. Feature Extraction

In EEG studies, particular ranges of neural oscillation in alpha (8–13 Hz) and beta (14–25 Hz) were shown to be associated with motor control and their applications to BCI have been investigated.

As MI has limited spatial resolution, low signal-to-noise ratio (SNR) and highly dynamic characteristics, the extraction of robust features is a crucial step in developing a successful BCI system. The common spatial patterns (CSP) algorithm was the most frequently used method in BCI system, which was introduced by Koles *et al.* in 1990 [49]. CSP method has been applied in MI hand movements [50]–[52] and MI foot movement [53]. CSP method maximizes the variance of signals for one class while minimizing the variance of the signals for the other. There are several methods to extend the CSP method to improve the classification accuracy, such as a filter bank common spatial pattern (FBCSP) [54], sparsity approach [55], [56], a sparse filter band common spatial pattern (SFBCSP) [57], temporally constrained sparse group spatial pattern (TSGSP) [58]. Therefore, we used the CSP feature as one feature, as the CSP feature is suitable for MI classification.

Except the traditional CSP feature for MI, another feature entropy can quantify the complexity and detect dynamic change through taking into account the non-linear behavior of time series [59]. Many approaches about entropy were applied to physiological signals. Approximate entropy (ApEn) presented by Pincus (1991) [60] is useable to quantify regularity in data without knowledge about a system, but it depends heavily on the record length. Sample Entropy (SampEn) proposed by Richman *et al.* (2000) [61] is an improvement of ApEn with respect to computation and accuracy of signal regularity. The advantage of SampEn is that it is insensitive to missing data. Permutation entropy (PermuEn) presented by Zanin *et al.* [62] has the quality of simplicity, robustness and very low computational cost. We also used Permutation disalignment index (PDI) [63] as an indirect, EEG-based, measure of brain connectivity to check the effect before and after massage therapy.

### E. Classifiers

We used support vector machine (SVM) [64] and logistic regression (LR) [65] for classification. SVM shows good performance in solving problems like small sample size, as well as being capable of both non-linear and high-dimensional pattern recognition. In SVM classifier, we used the Gaussian or radial basis function (RBF) kernel, and set the regularization parameter C=100, and gamma=0.001 in SVM. LR is a discriminative learning classifier that directly estimates the parameters of the posterior distribution function [65]. We used L1 and L2 regularization jointly to cope with the overfitting problem [66].

The other method was to apply CNN and attention-based bi-directional long short-term memory (BiLSTM). A 10-fold cross-validation method was used for validation of training set. Training set was set to 0.75 of the whole datasets, and the test set was set to 0.25 of the whole datasets. The parameter of random status was set to 7.

### F. Deep Learning Architecture

CNN has been used as the most commonly solution [67], as it outperformed other deep learning method in feature extraction. The CNN structure has many variations, such as LeNet-5, GoogleLeNet, residual neural network (ResNet), AlexNet, and the Visual Geometry Groupnetwork (VGGNet) [67]. VGGNet has been applied in EEG sleep patterns signals research. The main features of VGGNet include three points: the convolutional layer is followed by the max pooling layer to reduce the dimension, the number of convolution kernels is gradually increasing, and the convolutional layer stacking is used [68]. Considering VGGNets’ simplicity, we selected an improved VGGNet network in this article.

**FIGURE 4.**
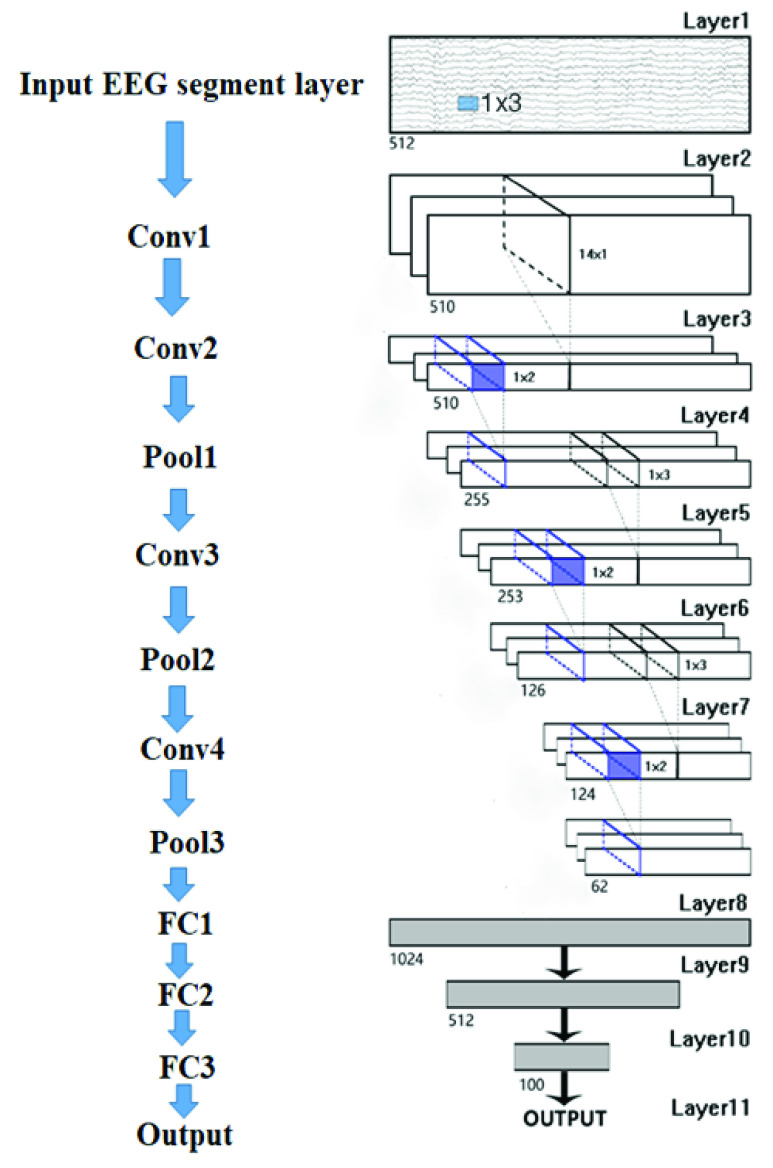
The architecture of the proposed CNN model. Conv indicates the convolutional layer, Pool indicates the pooling layer, FC indicates the fully connected layer. There are four convolutional layers (Conv1, Con2, Conv3, Conv4), three pooling layers (Pool1, Pool2, and Pool3) and three fully connected layers (FC1, FC2, and FC3).

We used two methods for the CNN method (one-dimensional convolution kernel and two-dimensional convolution kernel). First of all, we used one-dimension CNN, and it consists of 11 layers (Fig. 4). There are four convolutional layers (Conv1, Con2, Conv3, Conv4), three pooling layers (Pool1, Pool2, and Pool3) and three fully connected layers (FC1, FC2, and FC3). The input shape of the model was 7168 ($512\times 14$), where 512 was the number of temporal samples, and 14 was the number of channels. The four convolutional layers used 30 one-dimensional convolution kernels with a size of $1\times 3$ for the first convolutional layer, 25 kernels with a size of $14 \times 1$ for the second convolutional layer, 25 kernels with a size of $1\times 3$ for the third convolutional layer, and 50 kernels with a size of $1\times 3$ for the fourth convolutional layer. In all convolutional layers, the stride was 1 and the activation function was a linear rectification function (ReLu). After the first two convolutional layers, all the feature maps corresponding to the samples are one-dimensional, and these feature maps characterized the correlation and time characteristics between the different channels. Maximum pooling layers (Pool1, Pool2, and Pool3) were set with the pooling size $1\times 2$, and the step size 2. The number of hidden units for the fully connected layers (FC1, FC2, and FC3) were 1024, 512, 100, respectively. The softmax output layer completed the output of prediction results. Table 2 showed the different parameter values evaluated for CNN optimization.

**TABLE 2:** Different Parameter Values Evaluated for CNN Optimization

Parameters	Values/Types
Number of convolution layers	1, 2, 3, 4
Number of pooling layers	1, 2, 3
Number of fully connected layers	1, 2, 3
Number of convolutional filters	30, 25, 25, 50, 50, 100,100
Dimension of convolution filters	$1\times 3$, $14\times 1$, $1\times 3$, $1\times 3$
Activation functions	ReLu, softmax
Dropout probability	0.75
Maximum number of iterations	10000
Learning rate	$10^{-4}$

We also used the VGGnet with two-dimensional convolution kernel for comparison with the one-dimensional convolution network. the first convolution layer contained the $3\times 3$ kernel. In the second convolutional layer, the kernel size was $3\times 3$, with 32 input and 32 channels output. In the third convolutional layer, we adopted the kernel size $3\times 3$, 64 input and 64 output. In the fourth layer, we adopted maximum pooling layer with $2\times 2$ kernel and $2\times 2$ strides. In the fifth convolution layer, we adopted $3\times 3$ convolutional kernel, 64 input, 64 output and stride 1. In the sixth convolutional layer, we adopted $3\times 3$ kernel, 128 input, 128 output and stride 1. Then, the second maximum pooling layer was used with $2\times 2$ pooling size and $2\times 2$ step. Next, it goes through the flatten layer, the size is $4\times 7\times 128$. There were three fully connected layers in the end (Table 2). The results of VGGnet were similar to the proposed CNN model.

**FIGURE 5.**
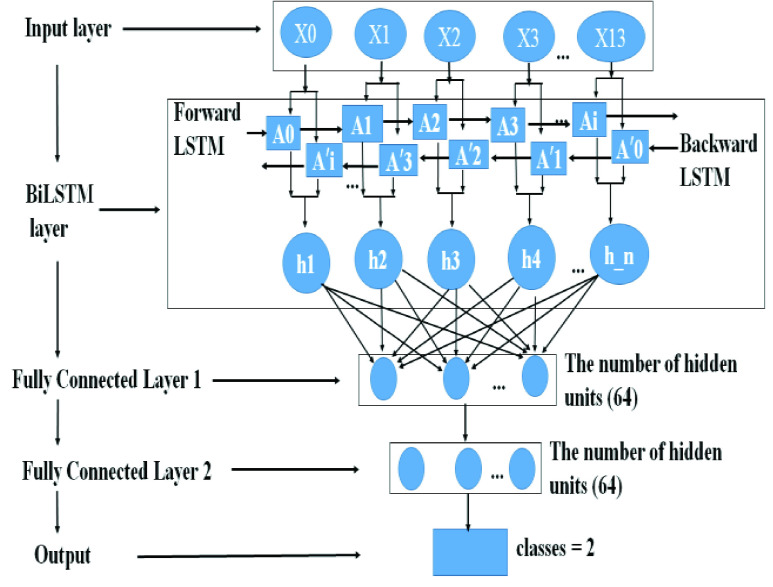
The architecture of the BiLSTM model.

Another deep learning method Bidirectional long short-term memory networks (LSTM) (Fig.5) was used in this study. LSTM introduced by Hochreiter & Schmidhuber (1997) is a special kind of recurrent neural network (RNN) architecture with long short memory units as hidden units. LSTMs are explicitly designed to avoid the long-term dependency problem. BiLSTM is composed of two ordinary LSTMs, a forward LSTM using past information, and a reverse order LSTM using future information, so that at time t, both the information at time t-1 and the information can be used Information to time t + 1. Generally speaking, since the bidirectional LSTM can use the information of the past time and the future time at the same time, the final prediction will be more accurate than the unidirectional LSTM. Table 3 showed the architectural details of the proposed BiLSTM model. As shown in the Fig.5, the number of LSTM neurons propagated forward and backward is 256. Data were input into BiLSTM network, then two fully connected layers were used. The number of neurons in the first and the second full connected layer was 64 and 64, respectively. In our study, the parameters were set as follows. Input layer included 14 channels, maximum length of series was 512 points, LSTM model size was 256, attention size was set to 8, batch size was set to 64, and hidden layer was set to 64 (Table 3).

**TABLE 3:** Architectural Details of the Proposed BiLSTM Model

Layers No.	Layer	Hyperparameter Details
0	Input layer	$14\times 512$
1	BiLSTM	256
3	Dense	64
4	Dense	64
5	Output layer	2

CNN and BiLSTM methods were implemented using Python, and the simulations were run on a computer with Windows 10 system with 32 GB of memory, a 256 GB Solid State Drives (SSD), an NVIDIA GeForce RTX 2070 card and a 8-core Intel (R) Core (TM) i7-9700 CPU @3.00 GHz.

### G. Statistical Analysis

Data were analyzed using Statistical Product and Service Solutions (SPSS) 19.0 software (IBM corporation, USA) to analyze the significant difference between two different conditions. The one-way analysis of variance (ANOVA) [69], [70] was used in analyzing the significant difference between two statuses. The significant difference p was set to 0.05. Repeated measures ANOVA compared outcome variables at baseline (measures taken immediately before the first massage) with outcome measures (measure taken immediately after massage). If the variance of two groups was not equal, we will choose nonparametric tests (Brown-Forsythe or Welch) to test the significance between two groups. We also used the Mann-Whitney U test when the variance was not equal for two groups. Mean value and standard deviation (SD) were plot in the figures.

## III. Results

### A. The Comparison Between the Original Signal and the Signal After MEMD-CCA

**FIGURE 6.**
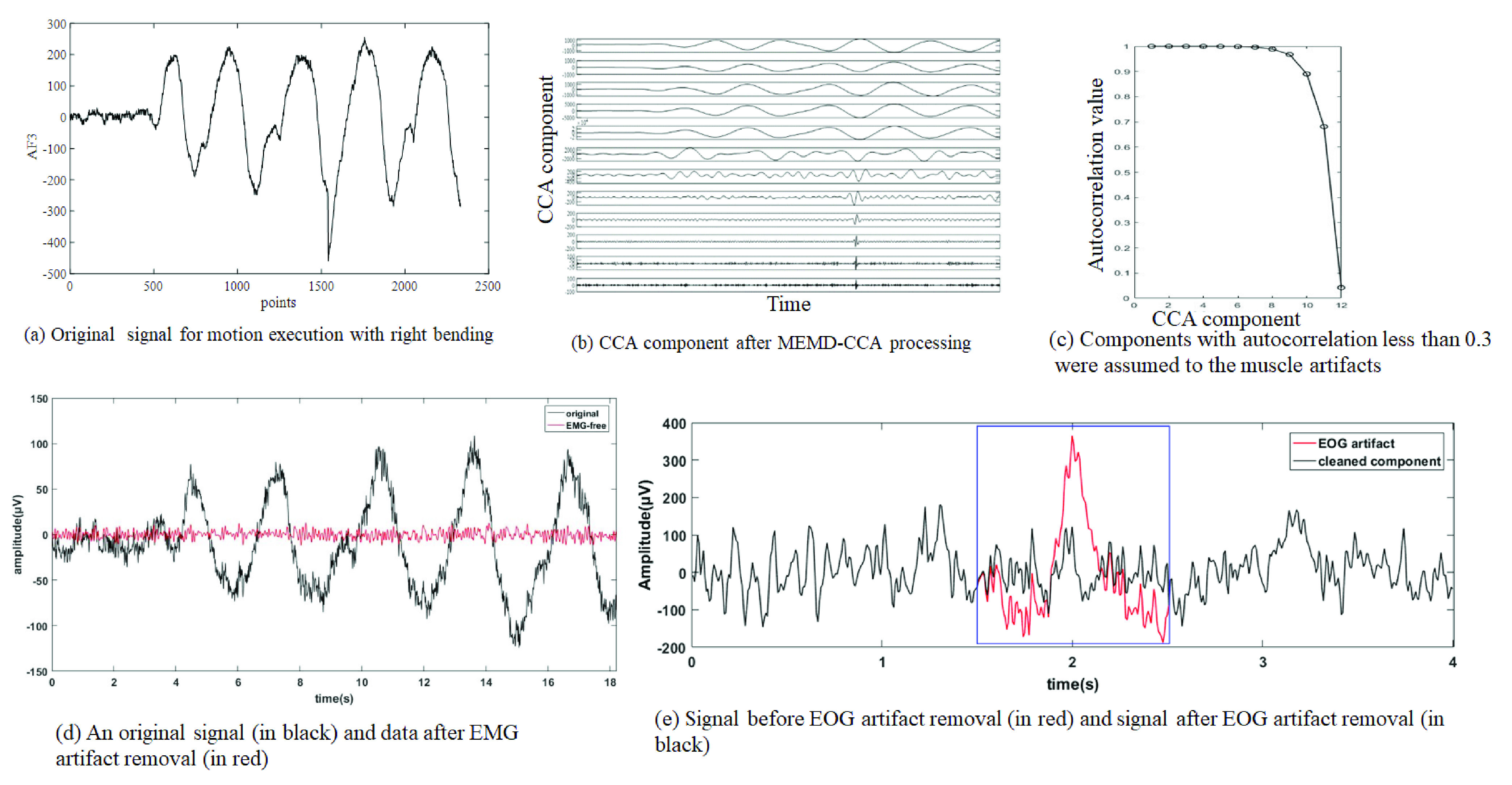
Artifacts removal. (a) Original signal of motion execution with right bending. (b) CCA component after MEMD-CCA processing for the AF3 channel. (c) Components with autocorrelation less than 0.3 were assumed to the muscle artifacts. (d) The original signal before (in black) and after EMG artifact removal (in red). (e) Signal before (in red) and after EOG artifact removal (in black).

Fig. 6 (a), (b) and (c) showed the original data of real right bending, the result after MEMD-CCA processing for the AF3 channel and the autocorrelation value of CCA component which was used in the EMG artifact removal. The signal after EMG artifact removal and the signal after EOG artifact removal were shown in Fig.6 (d) and (e). EMG and EOG artifact removal had a good performance.

### B. The Comparison Between the Power Spectrum Density of the Original Signal and the Signal After MEMD-CCA

**FIGURE 7.**
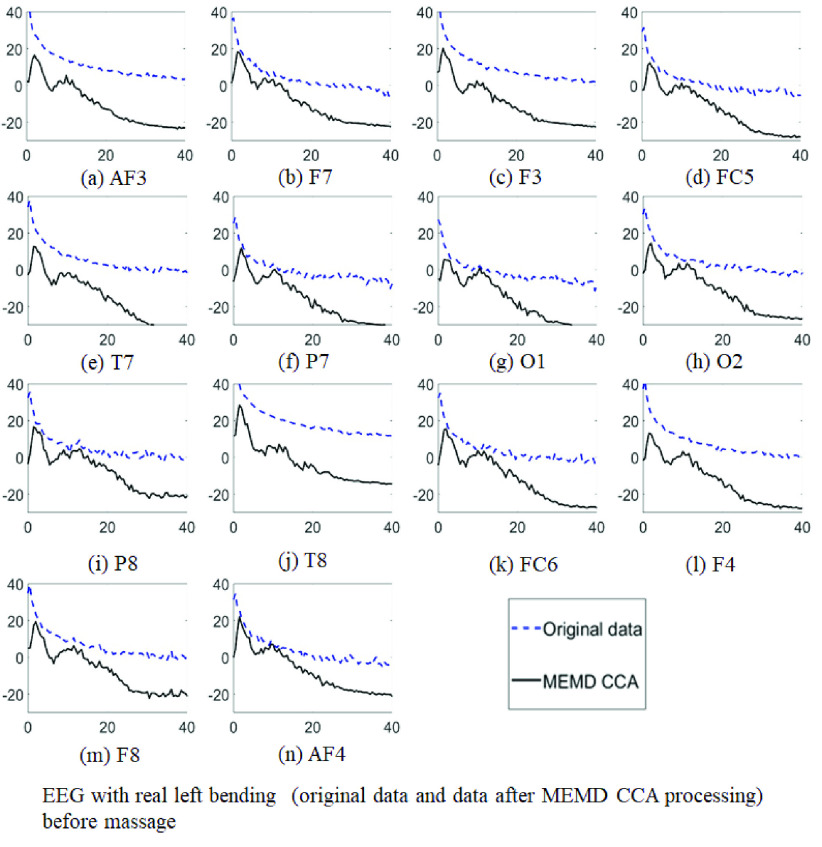
PSD of the original EEG data and the data after MEMD-CCA processing for ME with left bending before massage. The horizontal axis represents frequency with unit Hz and the vertical axis represents PSD with unit dB. (a) AF3. (b) F7. (c) F3. (d) FC5. (e) T7. (f) P7. (g) O1. (h) O2. (i) P8. (j) T8. (k) FC6. (l) F4. (m) F8. (n) AF4.

Fig. 7 showed the power spectrum density (PSD) of the original EEG data and the data after MEMD-CCA processing for EEG with real left bending before massage respectively. The fourteen channels were AF3, F7, F3, FC5, T7, P7, O1, O2, P8, T8, FC6, F4, F8 and AF4. The PSD values of the signal after MEMD-CCA in different channel were relatively lower in the high frequency band than the PSD in the low-frequency band, since the channels corrupted with heavy artifact in high frequency. These results were consistant with the results in [44] using the CCA method.

### C. The Comparison of SampEn of Four Rhythms Before and After Massage for Patients in Different Status

We calculated the statistical analysis of different features of alpha rhythm (mean±std) in Table 4. It was shown that the SampEn and PermuEn values were lower after massage than that before massage significantly.

**TABLE 4:** The Statistical Analysis of Different Features in Alpha Rhythm (Mean ± std)

Status	Features	rhythm	Channels with significant difference	Status 1 (Before massage) (mean ± std)	Status 2 (After massage) (mean ± std)	Significance (one-way ANOVA and nonparametric test)
Quiet	SampEn	alpha	AF3	0.6757 ± 0.0845	0.6668 ± 0.0800	p=0.002
			F7	0.6753 ± 0.0837	0.6682 ± 0.0830	p=0.014
			FC5	0.6757 ± 0.0847	0.6700 ± 0.0767	p=0.040
			T7	0.6950 ± 0.0835	0.6876 ± 0.0872	p=0.012
	PermuEn	alpha	AF3	1.2692 ± 0.0382	1.2652 ± 0.03565	p<0.001
			F7	1.2683 ± 0.0381	1.2659 ± 0.0356	p=0.032
			FC5	1.2692 ± 0.0377	1.2665 ± 0.0343	p=0.014
			O2	1.2697 ± 0.0370	1.2669 ± 0.0355	p=0.012
			AF4	1.2691 ± 0.0368	1.2663 ± 0.0360	p=0.012
MI with left bending	SampEn	alpha	AF3	0.6725 ± 0.0795	0.6648 ± 0.0749	p=0.028
			F3	0.6682 ± 0.0767	0.6606 ± 0.0764	p=0.030
	PermuEn	alpha	F7	1.2668 ± 0.0290	1.2647 ± 0.0327	p=0.004
			FC5	1.2684 ± 0.0294	1.2663 ± 0.0322	p=0.013
			O2	1.2699 ± 0.0308	1.2682 ± 0.0336	p=0.044
MI with right bending	SampEn	alpha	AF3	0.6653 ± 0.0739	0.6548 ± 0.0713	p=0.001
			F3	0.6645 ± 0.0730	0.6527 ± 0.0706	p=0.002
			FC5	0.6710 ± 0.0766	0.6603 ± 0.0728	p <0.001
			FC6	0.6799 ± 0.0748	0.6702 ± 0.0728	p=0.001
			AF4	0.6659 ± 0.0710	0.6579 ± 0.0701	p=0.008
	PermuEn	alpha	AF3	1.2674 ± 0.0310	1.2616 ± 0.0297	p <0.001
			F3	1.2658 ± 0.0303	1.2608 ± 0.0290	p < 0.001
			FC5	1.2683 ± 0.0316	1.2634 ± 0.0312	p =0.001
			FC6	1.2715 ± 0.0301	1.2676 ± 0.0312	p =0.001
			AF4	1.2658 ± 0.0296	1.2634 ± 0.0302	p =0.019
ME with left bending	SampEn	alpha	F7	0.6825 ± 0.0783	0.6751 ± 0.0789	p=0.012
			F3	0.6811 ± 0.0880	0.6698 ± 0.0893	p=0.002
			T7	0.6962 ± 0.0869	0.6824 ± 0.0926	p=0.014
			FC6	0.7018 ± 0.0849	0.6909 ± 0.0908	p=0.001
			AF4	0.6865 ± 0.0773	0.6780 ± 0.0768	p=0.01
	PermuEn	alpha	AF4	1.2752 ± 0.0313	1.2722 ± 0.0326	p =0.013
ME with right bending	SampEn	alpha	FC5	0.6921 ± 0.0882	0.6841 ± 0.0848	p=0.025
	PermuEn	alpha	FC6	1.2817 ± 0.0348	1.2868 ± 0.0370	p =0.002
Clinical assessment tool	VAS	-	-	4.6 ± 1.5	2.6 ± 1.8	p <0.01

MI = motion imagery, ME = motion execution, SampEn= Sample Entropy, PermuEn =Permutation Entropy, CSP = common spatial pattern, VAS=Visual analogue scale.

**FIGURE 8.**
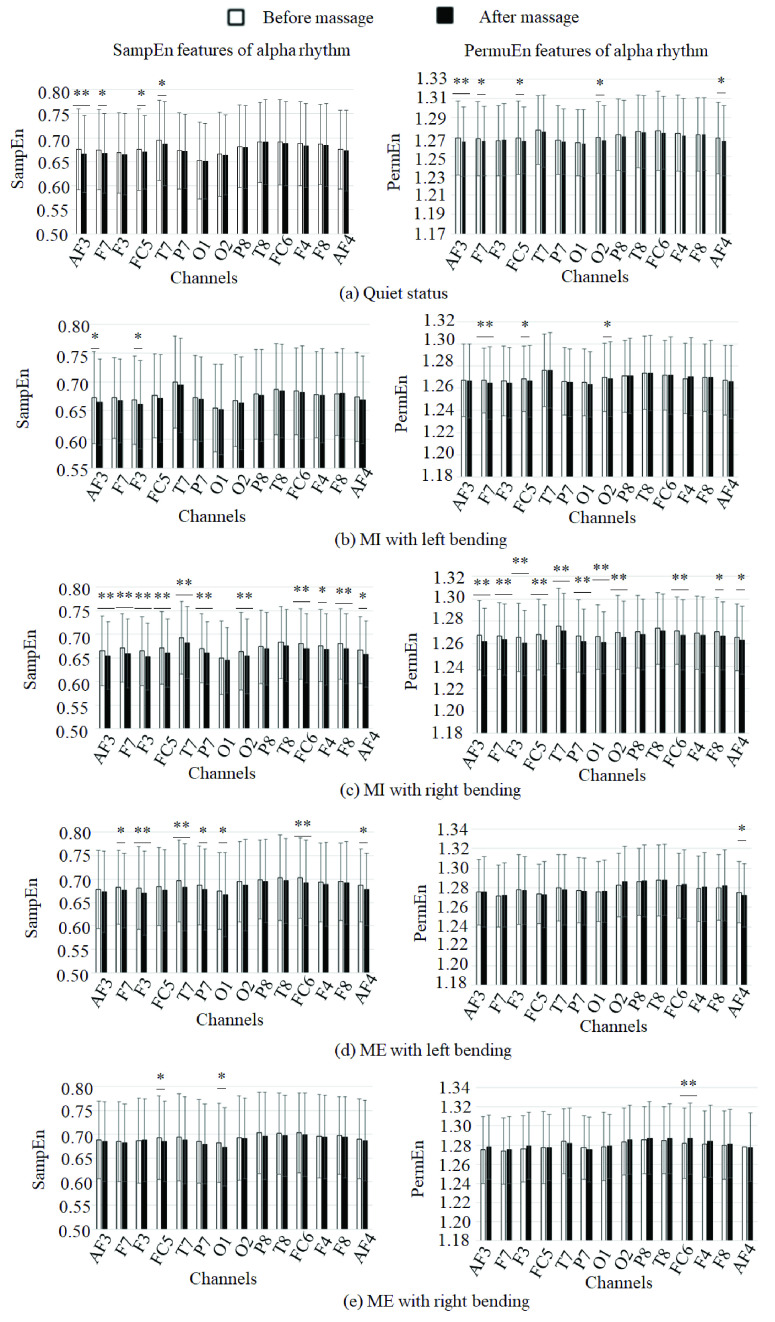
SampEn and PermuEn features of alpha rhythm before and after massage for patients in different status. (a) Quiet status. (b) MI with left bending. (c) MI with right bending. (d) ME with left bending. (e) ME with right bending. * denotes significant difference p<0.05. ** denotes significant difference p<0.01.

Fig. 8 showed that SampEn and PermuEn features of alpha rhythm decreased significantly after massage in almost fourteen channels than that before massage for patients in five statuses (quiet, MI with left/right bending, ME with left/right bending). At the same time, VAS was significantly lower after massage than before massage (Table 4). It indicates that massage alleviated the pain for the patients of skeletal pain, and it affects five statuses which showed the similar tendency.

**FIGURE 9.**
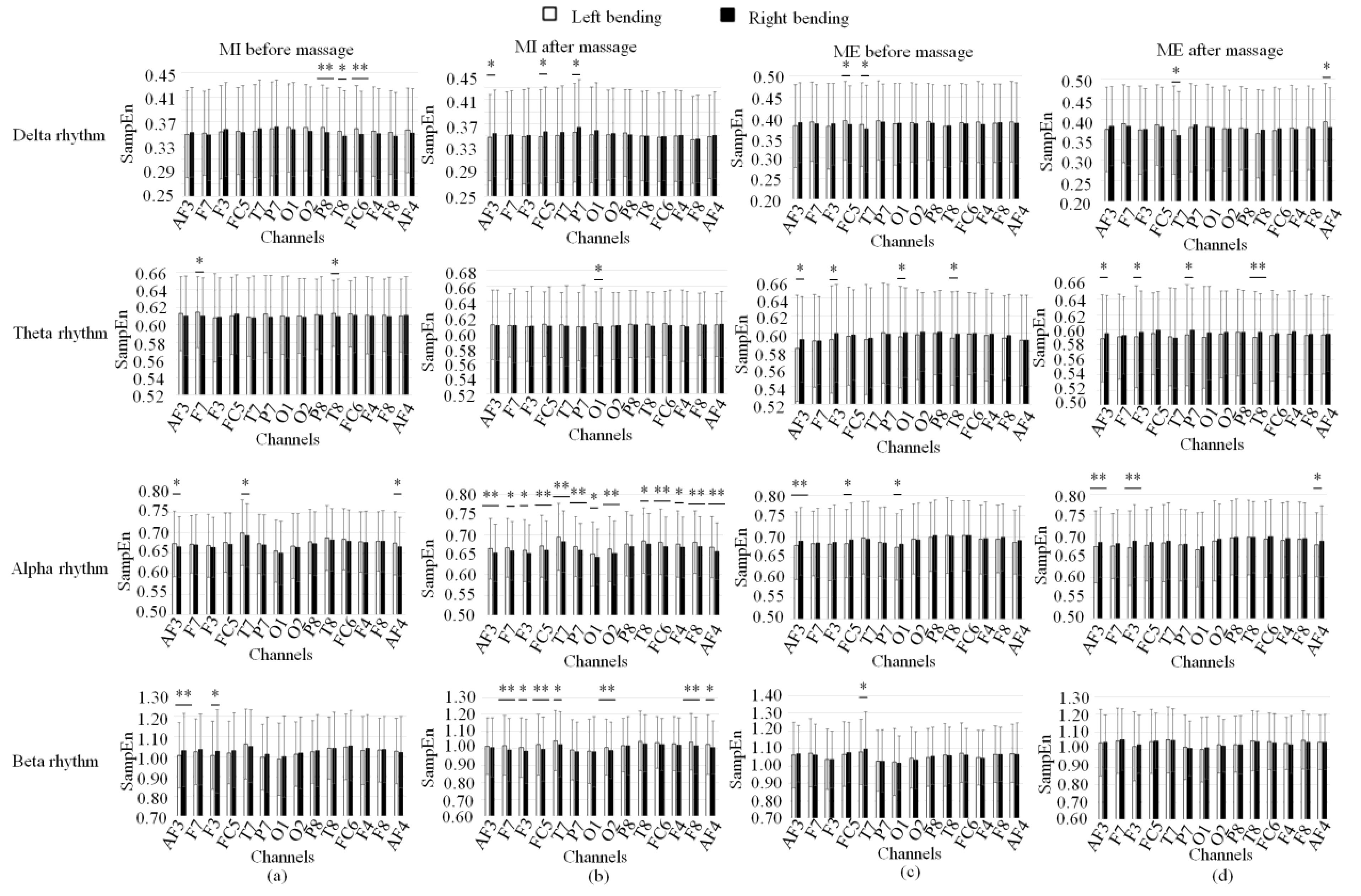
SampEn of four rhythms for patients doing left bending and right bending in four statuses. (a) Motion image (MI) before massage. (b) MI after massage. (c) Motion execution (ME) before massage. (d) ME after massage. * denotes significant difference p<0.05. ** denotes significant difference p<0.01.

Fig.9 showed the averaged SampEn of four rhythms for patients doing left bending and right bending in four statuses. It can be seen that there were increased channels with significant difference in alpha and beta rhythms for the MI with left bending and right bending (Fig. 9 (a) and (b)) after massage than that before massage. However, for ME after massage, there is no increased channels with significant difference for four rhythms of left and right bending when compared to the ME before massage (Fig. 9 (c) and (d)).

**FIGURE 10.**
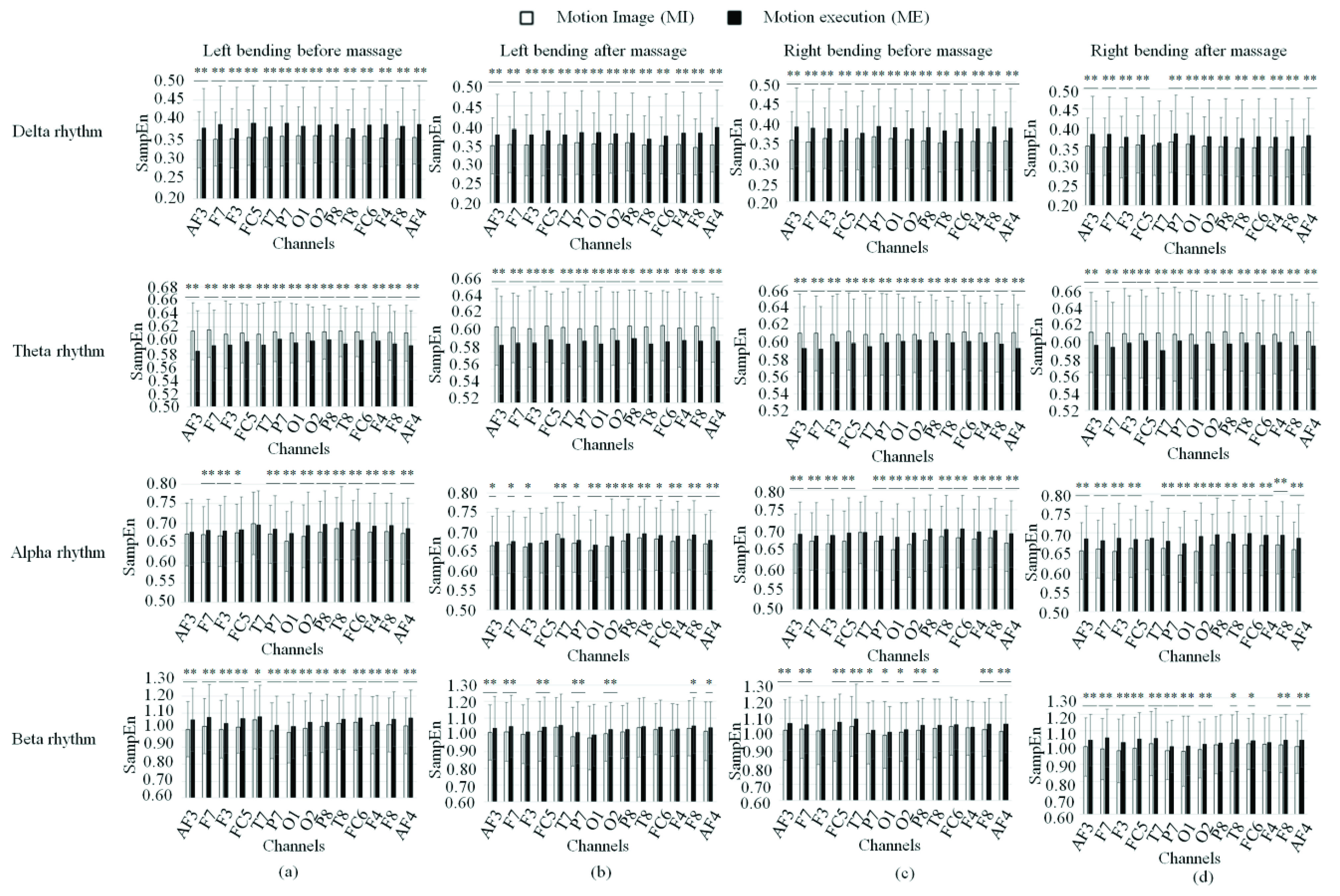
SampEn of four rhythms for patients doing MI and ME in four statuses. (a) Left bending before massage. (b) Left bending after massage. (c) Right bending before massage. (d) Right bending after massage. * denotes significant difference p<0.05. ** denotes significant difference p<0.01.

Fig.10 showed the averaged SampEn of four rhythms for patients doing MI and ME in four statuses. It can be seen that for four statuses (left bending before and after massage, right bending before and after massage), the averaged SampEn of delta, alpha and beta rhythm for the ME was significantly higher than that for the MI, and the averaged SampEn of theta rhythm for the ME was significantly lower than that for the MI.

### D. The Comparison of SampEn Topomaps of Four Rhythms Before and After Massage for Patients in Different Statuses

**FIGURE 11.**
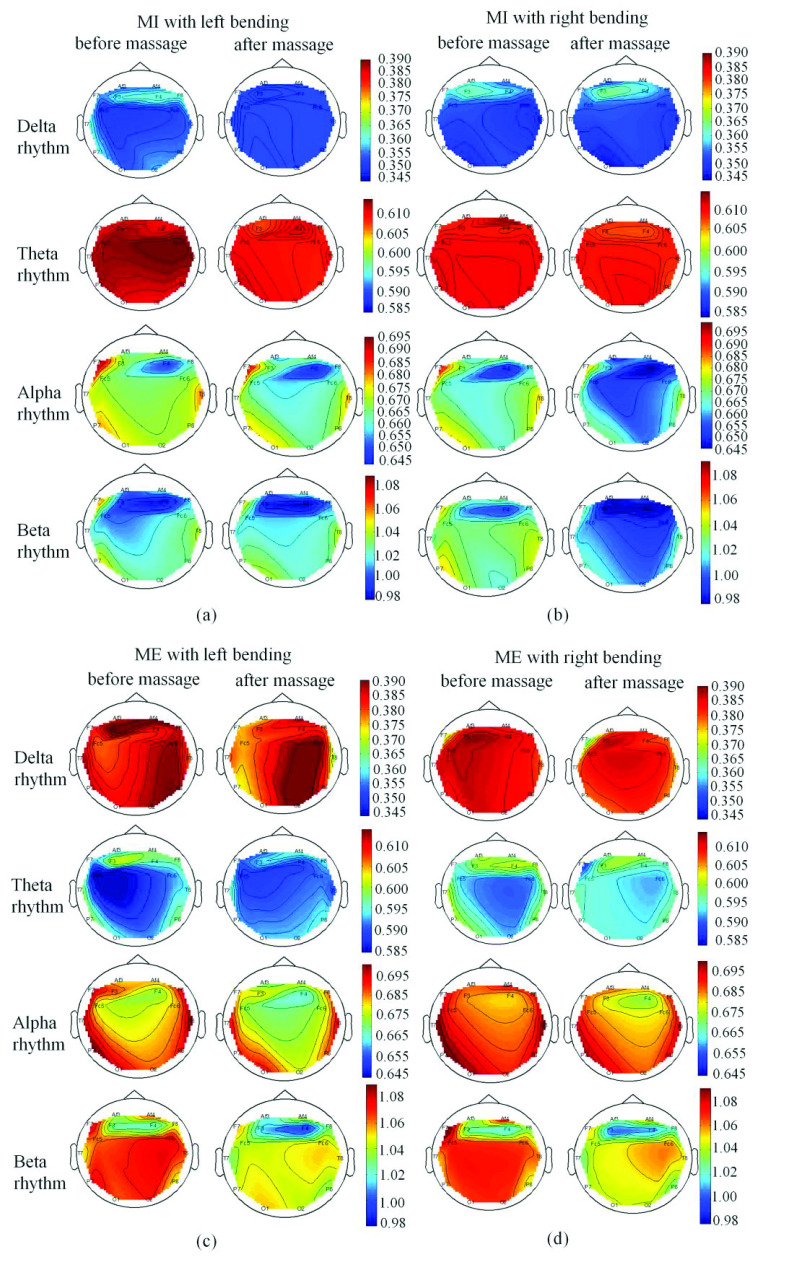
Patients’ four rhythms’ topomaps during (a) MI with left bending (before vs. after massage), (b) MI with right bending (before vs. after massage), (c) ME with left bending (before vs. after massage), (d) ME with right bending (before vs. after massage).

**FIGURE 12.**
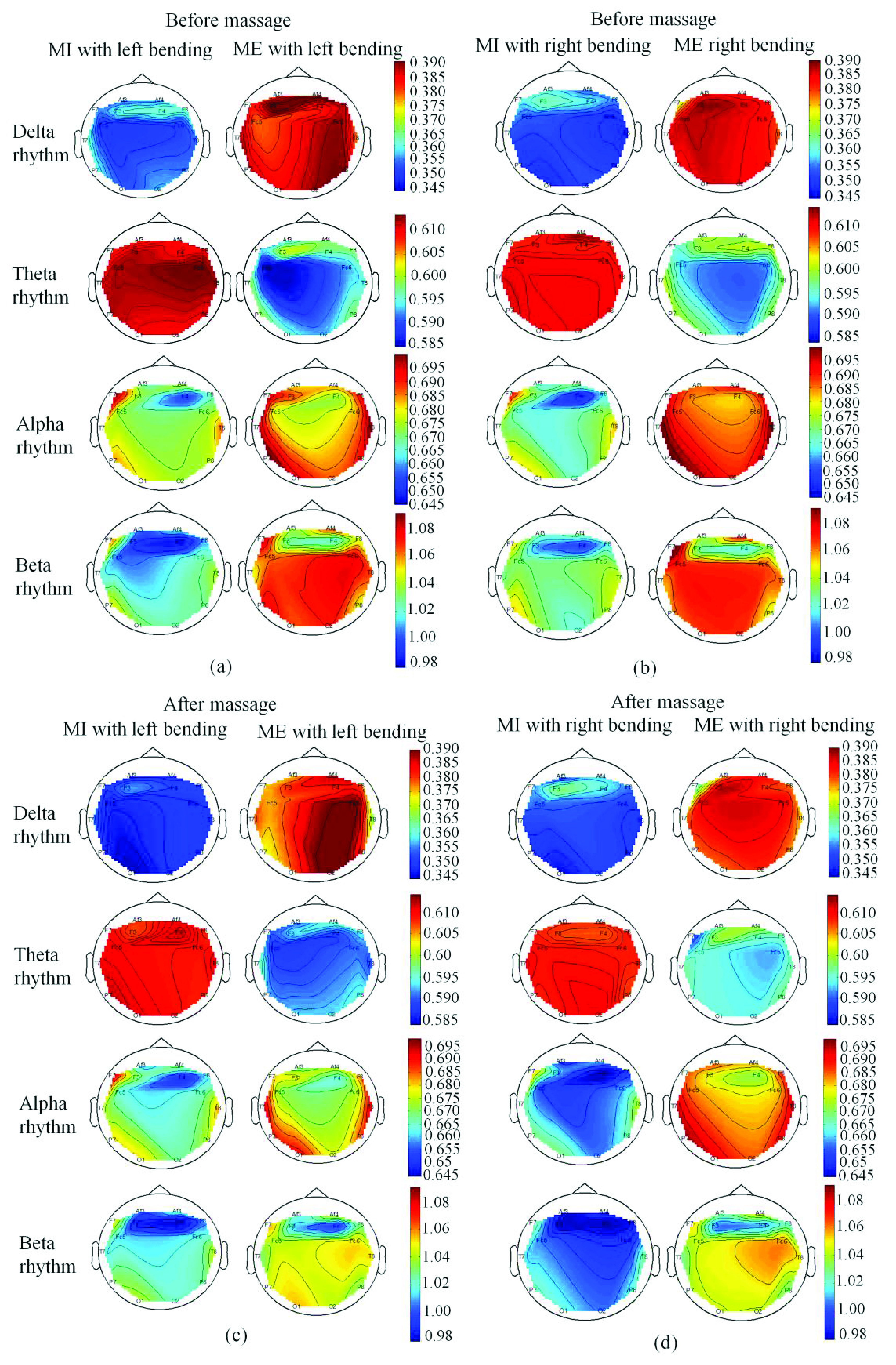
Patients’ four rhythms’ topomaps during (a) MI vs. ME with left bending before massage, (b) MI vs. ME with right bending before massage, (c) MI vs. ME with left bending after massage, (d) MI vs. ME with right bending after massage.

Brain topography analysis aimed to explore the brain connectome using either functional or effective connectivity during MI and ME tasks. Fig. 11 showed the comparison of SampEn topomaps of four rhythms before and after massage for patients in different statuses (MI with left bending, MI with right bending, ME with left bending, and ME with right bending). It can be seen that the averaged SampEn values in topomaps became smaller in four rhythms for MI with left bending after massage (Fig.11 (a)), and smaller SampEn values of three rhythms (theta, alpha, beta) could be found for MI with right bending after massage, ME left/right bending after massage (Fig. 11 (b)-(d)). Fig.12 showed the comparison between the MI and ME in different statuses. It can be seen that the SampEn values of the delta, alpha and beta rhythm were higher in ME than the MI with the same motion, while SampEn values of the theta rhythm were smaller in ME than the MI with the same motion.

### E. Performance Evaluation of Classifiers Using Different Features in Classifying Two Statuses (Before vs After Massage, MI vs Real Motions)

Table 6 showed Precision, F1-score, and Area under curve (AUC) (%) for the performance evaluation of classifiers in classifying two statuses (before and after massage). Table 7 showed Precision, F1-score, and AUC for the performance evaluation of classifiers in classifying two statuses (MI and real motions).

**TABLE 5:** Comparisons Between Two Statuses (Quiet, me, mi) Using the Data After Filtering (1–40 Hz) and Artifacts Removal of Participants (Mean ± std)

Comparisons	Methods	Accuracy (%)
Quiet vs. ME	CNN+LSTM	86.95 ± 0.79
Quiet vs. MI	CNN+LSTM	68.09 ± 4.32
MI vs. ME	CNN+LSTM	85.78 ± 1.14

**TABLE 6:** Precision, F1-Score, and Area Under Curve (AUC) (%) for the Performance Evaluation of Classifiers in Classifying Two Statuses (Before Massage vs. After Massage)

Features	Status (before massage vs after massage)	Performance evaluation	Classifiers							
			SVM				LR			
			Delta	Theta	Alpha	Beta	Delta	Theta	Alpha	Beta
CSP	MI with left bending	Precision	60(0)	60(0.5)	62 (2)	61 (2)	60(0)	59(1)	62 (1)	60(2)
		Recall	60(8)	60(11)	63 (1)	61 (2)	60(2)	59 (1)	62(1)	60(0.5)
		F1	60(4)	60(5)	62(0.5)	60(0.5)	60(4)	59 (1)	62 (1)	60(0.5)
		AUC	60	60	63	61	60	59	62	60
	MI with right bending	Precision	58(1)	58(1)	61(0.5)	60 (1)	57(1)	58(0.5)	62 (1)	60 (1)
		Recall	57(17)	58(4)	61(4)	60 (10)	57(6)	58 (4)	62 (3)	60 (3)
		F1	56(9)	58(2.5)	61(2.5)	59 (5)	56(4)	58 (3)	62 (2)	60 (2)
		AUC	57	58	61	60	57	58	62	60
	Real left bending	Precision	60(0)	60(0.5)	62(2)	60(1)	60(2)	57(4)	61(1)	59(2)
		Recall	57(25)	55(31)	57(30)	60(23)	58 (19)	56(15)	58 (20)	58 (18)
		F1	54(14)	50(20)	53(16)	57(13)	58 (10)	55(9)	57 (11)	58 (11)
		AUC	57	55	57	57	58	56	58	58
	Real right bending	Precision	57(1)	57(1)	60(0)	62(2)	57(2)	57(2)	60(1)	61(2)
		Recall	57(15)	57(10)	60(12)	62 (2)	57(6)	57(3)	59(6)	61(4)
		F1	56(8)	57(6)	60(6)	62 (2)	57(4)	57(2)	59(4)	61(3)
		AUC	57	57	59	62	57	57	59	61
SampEn	MI with left bending	Precision	53(1)	52(1)	51(1)	50(1)	51(1)	51(1)	49(2)	49(2)
		Recall	52(34)	52(20)	52(25)	50(24)	51(13)	51(13)	49(14)	50 (10)
		F1	46(19)	50(11)	48(14)	47(14)	50(8)	50(8)	48(8)	49 (6)
		AUC	51	52	51	50	51	51	49	49
	MI with right bending	Precision	45(4.5)	48(3)	53(1)	59(0.5)	45(3)	44(5)	53 (2)	53 (2)
		Recall	46(23)	49(27)	53(10)	58(17)	45(13)	45(22)	53 (1)	53 (5)
		F1	43(15)	45(15)	53(5)	57(9)	45(7)	43(15)	53 (1)	53 (4)
		AUC	45.9	47.4	54.2	61.0	45.4	46.1	52.7	55.4
	Real left bending	Precision	61(1.5)	50(7)	53(5)	58(4)	56(3)	58(0)	56(3)	51 (6)
		Recall	54(39)	50(45)	52(34)	57(19)	54(31)	52(43)	53 (31)	51 (31)
		F1	49(24)	40(30)	48(19)	56(12)	51(7)	44(28)	50 (20)	48 (20)
		AUC	54.4	49.9	52.7	56.6	54.5	51.1	56.6	50.9
	Real right bending	Precision	52(5)	49(5)	48(5)	52(5)	52 (4)	50(6)	50 (5)	51(5)
		Recall	52(3)	49(6)	48(3)	52(3)	52(13)	50(17)	50(14)	51(6)
		F1	52(4)	49(5)	48(1)	52(1)	51(11)	49(11)	50 (9)	51(5)
		AUC	52.2	49.0	49.8	52.3	51.6	50.6	50.1	51.2
PermuEn	MI with left bending	Precision	48(3)	56(2)	55(2)	65(1)	54(3)	53(3)	52 (2)	52 (2)
		Recall	48(4)	56(1)	55(10)	64(14)	53(11)	53(4)	52(12)	52(7)
		F1	48(3)	56(3)	54(6)	63(7)	53(7)	53(3)	51 (7)	52(4)
		AUC	48.4	55.5	54.4	63.5	54.5	53.5	53.7	54.4
	MI with right bending	Precision	55(2)	49(2)	55(2)	63(1)	54(1)	53(1)	54(2)	58 (1)
		Recall	55(4)	49(0.5)	55(3)	63(4)	53(15)	53(9)	54(7)	58(7)
		F1	55(3)	49(1)	55 (3)	63(2)	52(8)	52(6)	54(4)	58(4)
		AUC	55.1	49.3	55.2	63.1	53.6	56.2	56.9	59.3
	Real left bending	Precision	50(7)	50(6)	53(7)	55(6)	47(10)	49(8)	52(5)	54(5)
		Recall	50(14)	50(17)	53(12)	55(13)	49(28)	50(37)	51(33)	53(25)
		F1	49(10)	50(12)	53(9)	55(10)	42(26)	44(25)	48(21)	51(16)
		AUC	49.6	50.4	53.2	54.8	51.2	49.9	52.3	56.1
	Real right bending LDH	Precision	52(5)	53(5)	56(5)	54(5)	50(5)	52(4)	50(5)	55(4)
		Recall	52(8)	53(5)	56(2)	54(1)	50(10)	52(11)	50(13)	55(11)
		F1	52(6)	53(5)	56(4)	54(2)	50(8)	52(8)	50(9)	55(8)
		AUC	51.8	52.7	55.6	54.1	49.2	55.9	51.7	54.0

Note: CSP-common spatial pattern, SVM-support vector machine, PermuEn- permutation entropy. All data except for AUC are shown as mean±std.

**TABLE 7:** Precision, F1-Score, and Area Under Curve (AUC) (%) for the Performance Evaluation of Classifiers in Classifying Two Statuses (MI vs. ME)

Features	Statuses (motion image vs motion execution)	Performance evaluation	Classifiers							
			SVM				LR			
			Delta	Theta	Alpha	Beta	Delta	Theta	Alpha	Beta
CSP	Left bending before massage	Precision	68(2)	74(3)	69 (1)	62 (0)	69(1)	75(1)	71 (2)	67(0)
		Recall	68(5)	74(6)	69 (1)	62 (2)	68(10)	75 (2)	71(1)	67(7)
		F1	60(1)	73(2)	69(1)	62(1)	68(4)	75 (1)	71 (1)	67(4)
		AUC	68	74	69	62	68	75	71	67
	Left bending after massage	Precision	68(1)	71(0)	69 (1)	63(1)	68(1)	72(1)	69 (2)	61 (2)
		Recall	68(5)	71(6)	69 (6)	63 (9)	68(6)	72 (4)	69 (1)	61 (5)
		F1	68(3)	71(3)	69(3)	63 (5)	68(4)	72 (2)	69 (1)	61 (4)
		AUC	68	71	69	63	68	72	69	61
	Right bending before massage	Precision	68(1)	72(2)	72(3)	65(4)	69(0)	72(0)	71 (2)	64(2)
		Recall	68(7)	71(7)	72(5)	65(9)	69(4)	72 (3)	71(1)	64(3)
		F1	68(3)	71(2)	71(0.5)	64(2)	69(2)	72 (1)	71 (1)	64(1)
		AUC	68	71	72	65	69	72	71	64
	Right bending after massage	Precision	69(1)	75(1)	71(2)	67(0)	69(1)	75(0.5)	69 (2)	67 (2)
		Recall	68(10)	75(2)	71(1)	67(8)	69(5)	74 (4)	70 (1)	67 (2)
		F1	68(4)	75(1)	71(1)	67 (4)	69(3)	75 (3)	69 (1)	67 (2)
		AUC	68	75	71	67	69	74	70	67
SampEn	Left bending before massage	Precision	77(4)	67(1)	63(1)	58(1)	64(1)	68(3)	61(1)	56(1)
		Recall	77(11)	67(3)	63(6)	57(11)	64(1)	68(8)	61(5)	56 (1)
		F1	77(3)	67(1)	63(2)	57(6)	64(1)	68(3)	61(3)	56 (1)
		AUC	77	67	63	58	64	68	61	56
	Left bending after massage	Precision	71(2)	61(2)	66(2)	59(2)	69(1)	65(0)	56 (3)	52 (4)
		Recall	69(15)	61(9)	63(21)	59(13)	68(9)	63(15)	56 (14)	52 (16)
		F1	69(7)	61(6)	62(10)	58(7)	68(4)	63(8)	55 (8)	52 (10)
		AUC	69	61	62	58	69	63.4	55	52
	Right bending before massage	Precision	77(4)	64(1)	64(2)	62(3)	64(1)	65(2)	60(0)	58 (1)
		Recall	76(11)	64(5)	64(14)	61(5)	64(2)	65(11)	60 (9)	58 (7)
		F1	76(3)	64(3)	63(6)	61(1)	64(1)	64(5)	60 (4)	58 (4)
		AUC	76	64	64	62	64	64	60	58
	Right bending after massage	Precision	72(1)	63(2)	59(1)	62(3)	64(1)	63(1)	62 (1)	60(2)
		Recall	70(11)	62(10)	58(15)	62(4)	64(13)	62(12)	61(14)	60(8)
		F1	70(5)	62(6)	58(9)	62(4)	63(6)	62(6)	61(7)	60(5)
		AUC	70	62	58	61	63	62	61	60
PermuEn	Left bending before massage	Precision	84(3)	68(3)	64(0.5)	65(4)	83(1)	68(1)	61(1)	63(0)
		Recall	84(4)	68(9)	64(0.5)	63(17)	83(1)	68(0)	61(1)	63(3)
		F1	84(1)	68(3)	64(0.5)	62(7)	83(0)	68(0)	61(0.5)	63(1.5)
		AUC	84	68	64	63	83	68	61	63(1.5)
	Left bending after massage	Precision	85(4)	66(3)	64(1)	64(6)	83(1)	66(1)	63(0.5)	60(2)
		Recall	83(9)	66(3)	63(15)	63(10)	83(4)	65(7)	62(9)	60(8)
		F1	83(2)	66(2)	63(7)	62(2)	83(2)	66(5)	62(6)	60(5)
		AUC	83	66	63	63	83	65	62	60
	Right bending before massage	Precision	83(5)	68(0.5)	62(2)	67(5)	82(1.5)	68(0)	59(0)	64(0.5)
		Recall	81(10)	68(4)	60(17)	67(8)	81(4)	68(4)	59(10)	64(3)
		F1	81(2)	68(2)	59(8)	66(2)	81(1.5)	68(2)	59(5)	64(3)
		AUC	81	68	60	67	81	68	59	64
	Right bending after massage	Precision	82(6)	68(2)	67(2)	68(6)	79(0.5)	66(0)	65(0.5)	64(2)
		Recall	80(12)	68(0.5)	65(16)	67(8)	79(4)	65(8)	65(12)	63(5)
		F1	80(3)	68(1)	64(6)	67(1)	79(2)	65(5)	64(5)	64(3)
		AUC	80	68	65	67	79	65	65	63

Note: CSP-common spatial pattern, SVM-support vector machine. All data except for AUC are shown as mean±std.

### F. The Accuracy of Classification for MI With Trunk Left/Right Bending Using CNN and BiLSTM

**FIGURE 13.**
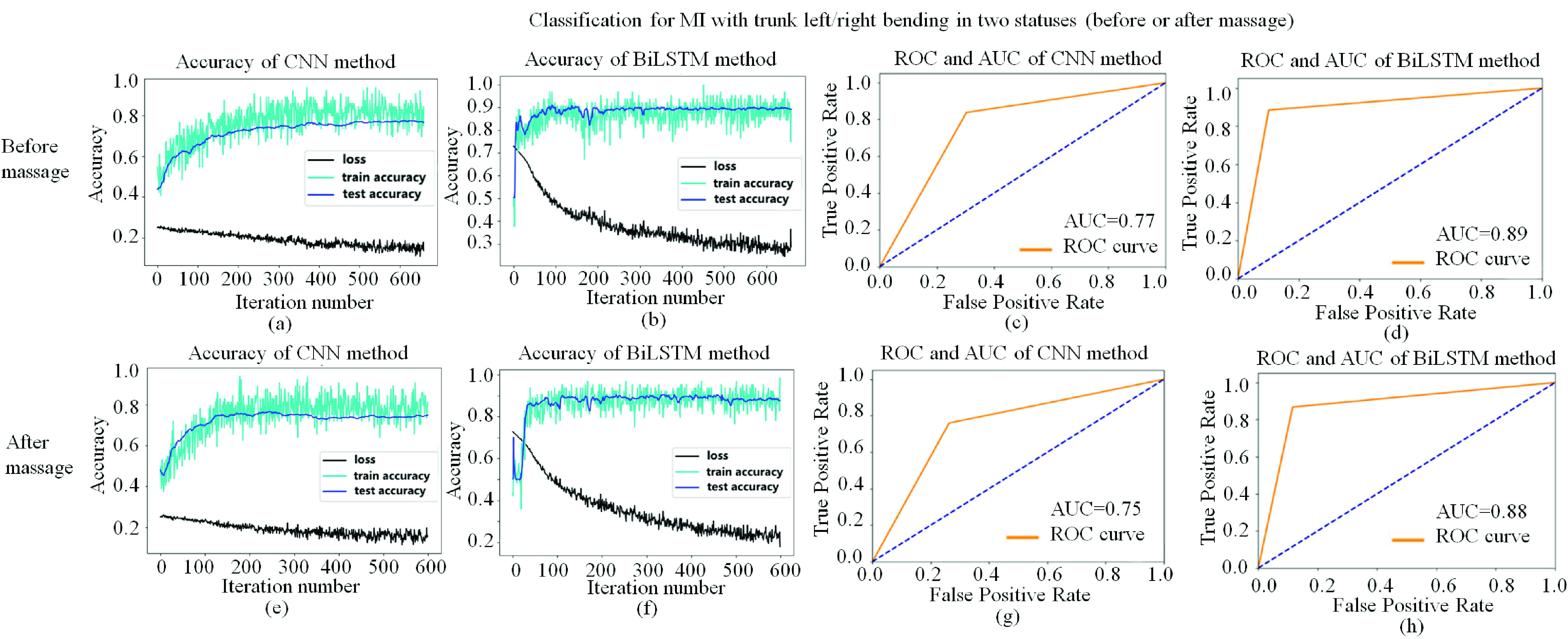
Performance of classification for MI with trunk left/right bending before massage using CNN and BiLSTM. (a) Accuracy of CNN method before massage. (b) Accuracy of BiLSTM method before massage. (c) Receiver operating characteristic (ROC) and Accuracy under curve (AUC) of CNN method before massage. (d) ROC and AUC of BiLSTM method before massage. (e) Accuracy of CNN method before massage. (f) Accuracy of BiLSTM method before massage. (g) ROC and AUC of CNN method before massage. (h) ROC and AUC of BiLSTM method before massage.

Fig. 13 showed the performance of classification for MI with trunk left/right bending using CNN and BiLSTM methods for the patients with skeletal muscle pain before or after massage therapy respectively. Before the massage therapy, the AUC for CNN and BiLSTM was 0.77 and 0.89, respectively. After the massage therapy, the AUC for CNN and BiLSTM was 0.75 and 0.88, respectively.

It was interesting that the averaged test accuracy decreased a bit using the patient’s data after massage therapy using both methods (CNN and BiLSTM). Furthermore, the AUC of BiLSTM was higher than that of CNN method in both cases (before and after massage therapy). It may due to that the massage therapy make the patients with skeletal pain feel more comfortable on the affected side and pain decreased. The difference of patients’ motor imagery between left and right bending decreased.

We also used the data after filtering (1–40) Hz, and combined all the data in quiet, ME, or MI statuses. Table 5 showed that using CNN+LSTM method. The accuracy of classification was 86.95±0.79%, 68.09±4.32, 85.78±1.14 for quiet vs. ME, quiet vs. MI, MI vs. ME, respectively.

### G. PDI Scores for Four Rhythms of Patients in 13 Inter- Channels

**FIGURE 14.**
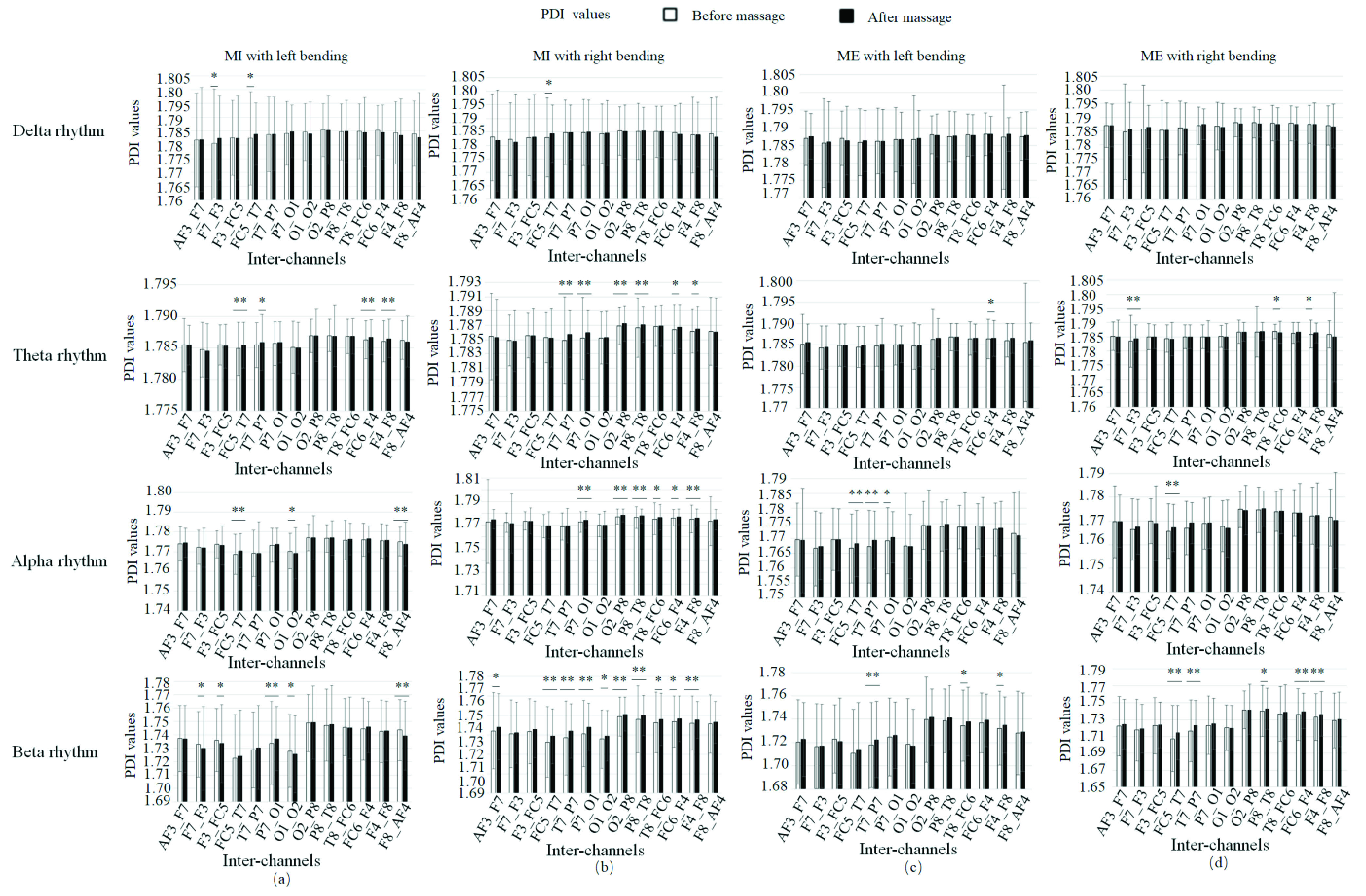
PDI values of four rhythms before and after massage for patients in different statuses. (a) MI with left bending. (b) MI with right bending. (c) ME with left bending. (d) ME with right bending. * denotes significant difference p<0.05. ** denotes significant difference p<0.01.

**FIGURE 15.**
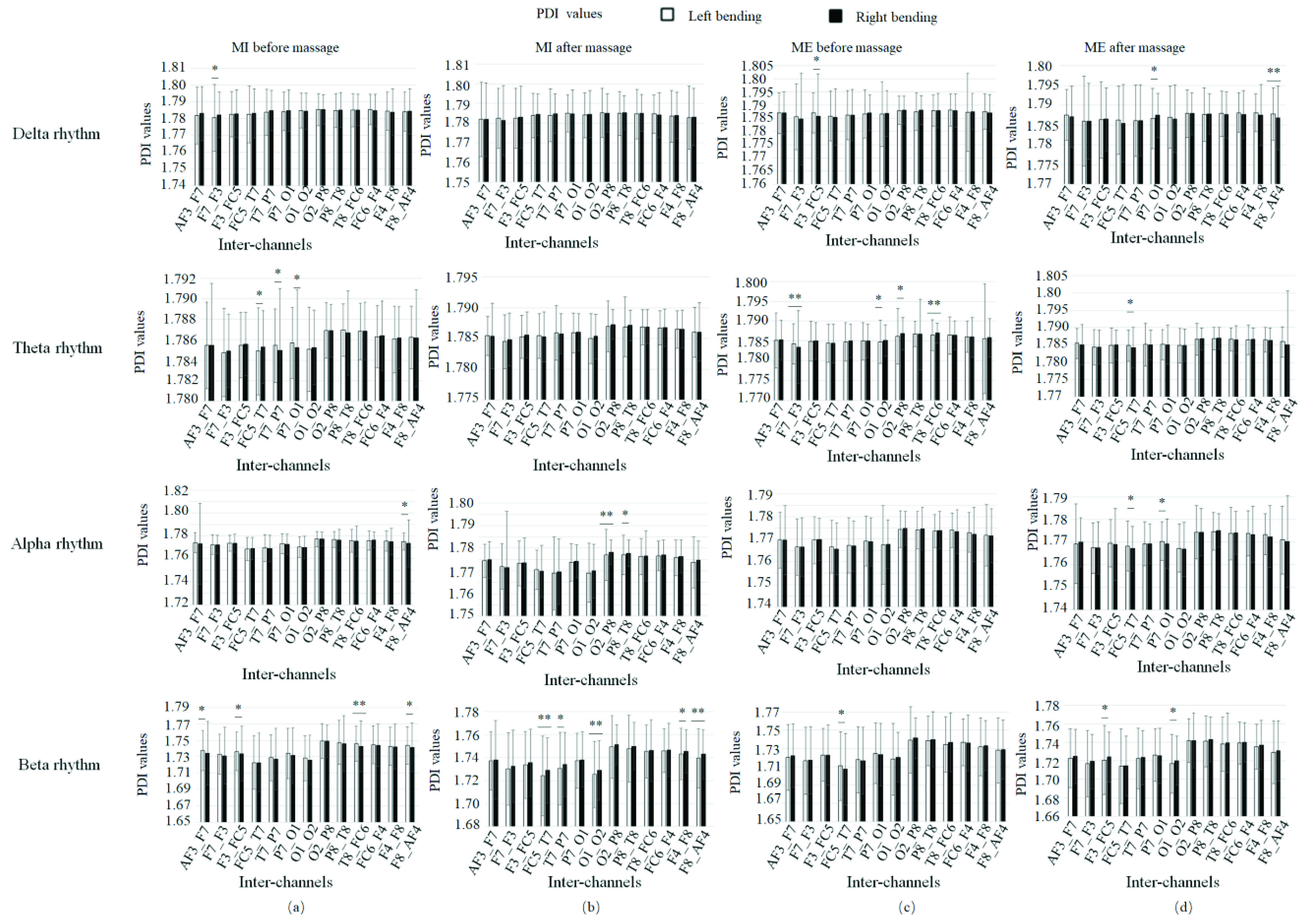
PDI values of four rhythms for patients doing left bending and right bending in different statuses. (a) MI before massage. (b) MI after massage. (c) Real motion before massage. (d) Real motion after massage. * denotes significant difference p<0.05. ** denotes significant difference p<0.01.

**FIGURE 16.**
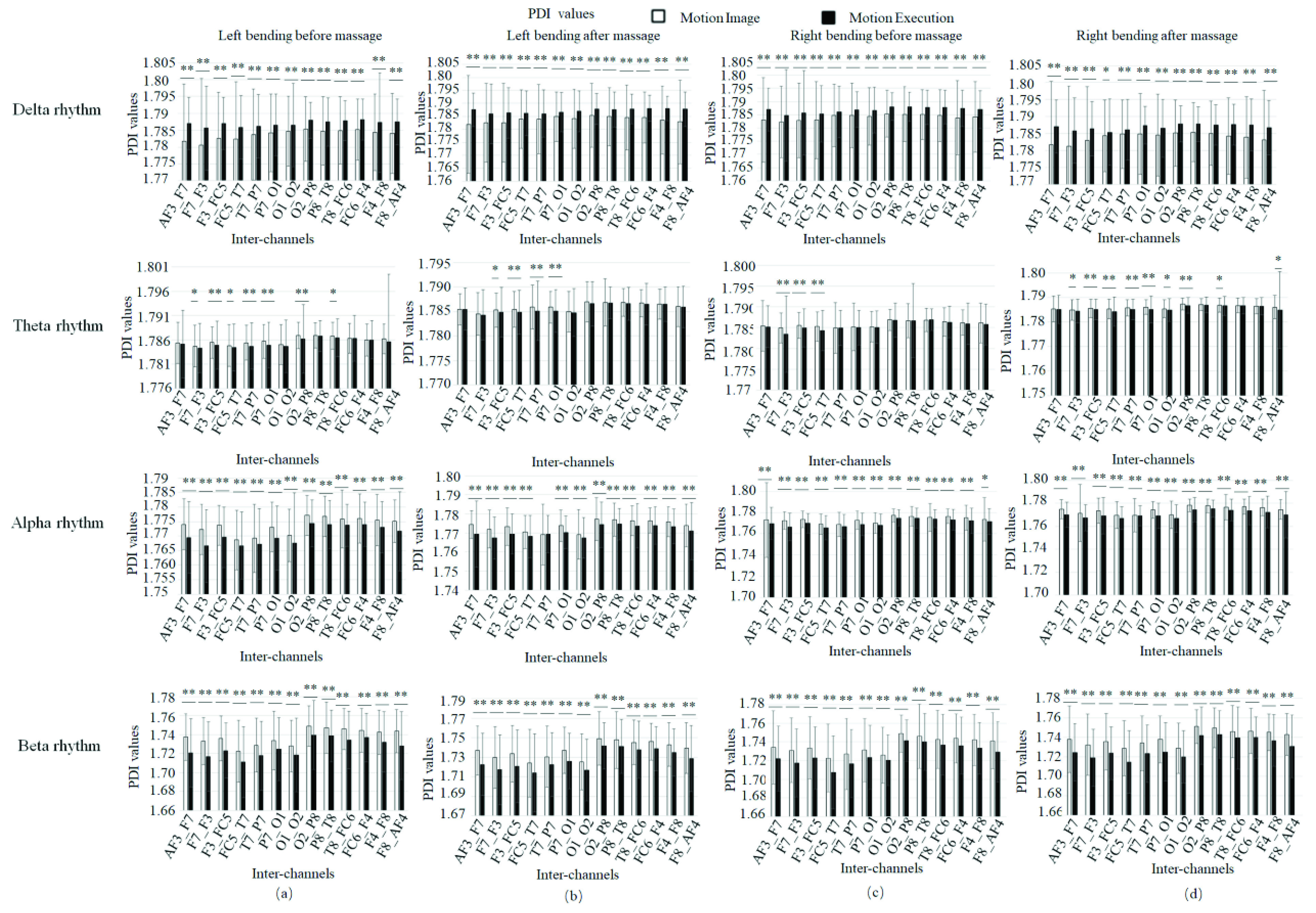
PDI values of four rhythms for patients doing MI or ME in different statuses. (a) Left bending before massage. (b) Left bending after massage. (c) Right bending before massage. (d) Right bending after massage. * denotes significant difference p<0.05. ** denotes significant difference p<0.01.

Fig. 14 showed the PDI scores before and after massage in 13 inter-channels for patients in different statuses. Fig.15 showed the PDI values of four rhythms for patients doing left bending and right bending. Fig.16 showed the PDI values of four rhythms for patients doing MI or ME in different statuses (left bending before massage, left bending after massage, right bending before massage and right bending after massage).

## IV. Discussion

The current study investigated the differences in brain activity variation resulting from massage for the MI with left/right bending and real left/right bending. We used SampEn, PermuEn and CSP for the feature extraction, and we used SVM and LR classifier for the classification. We also used the deep learning architecture (CNN and BiLSTM) for the classification of MI with left/right bending and real left/right bending. Fig.9 showed the differences of SampEn of four rhythms before and after massage for patients in ME and MI statuses for the first time. The SampEn and PermuEn features for MI with trunk bending showed significant decrease for the massage therapy effectiveness, which indicated the lower complexity in the left hemisphere. The complexity of the topomaps became less after massage than that before massage. These results were consistent with the decreased VAS for the clinical outcome. It indicated that the massage changed the relaxation level of patient.

Fig.14 also showed that there were significant differences using the PDI score in beta rhythm in four statuses after massage compared to the same motion before massage. This result was coincident with the result in [62] which showed that the hypnosis during real movement can significantly reduce ERD during motor performance.

Very few studies analyze connectivity patterns revealed by EEG during MI. Few of them focused on differences in connectivity patterns between MI and motor execution [12], [26], [71]. These motions include real and imaginary rhythmic for real foot, imaginary foot, real hand, and imaginary hand movements [12], [27] and finger tapping [71], swallowing [22], self-feeding activity using chopsticks [70]. ME and MI are very similar processes [70]. The studies found overlapping activity in the inferior frontal gyrus and precentral regions (including premotor areas and SMA) between both tasks [29]. Rimbert *et al.* [16] presented the hypnotic statuses modulated sensorimotor beta rhythms during ME and MI. It was suggested that the status of hypnosis changed the sensorimotor beta rhythm during the ERD phase but maintains the ERS phase in the mu and beta frequency band. In our study, it indicated a different activation of the motor cortex due to the massage therapy. To our knowledge, massage therapy’s effectiveness on the classification of MI and ME with left/right motions in patients with skeletal muscle pain has not been investigated.

Previous work [72] showed that the averaged decoding accuracy among all participants was only 67.1 ± 12.5% (mean±SD) for the ME, and decoding accuracy was 48.7 ± 8.7% (p<0.05) for the imagined movements. J. Asensio-Cubero *et al.* [73] applied wavelet lifting over graphs for EEG in BCI applications with the mean accuracy from 0.527 (± 0.08) to 0.624 (± 0.13). They used wavelet with LS that obtains better classification performance for 85% of the subjects studied than FGWs under the same conditions [73].

Daly *et al.* [26] showed that the averaged AUC was about 80% for the executed taps and imagined taps at each frequency band. Chaisaen *et al.* [25] showed that the classification of action observation and MI providing the highest mean accuracy at 82.73± 2.54% in the stand-to-sit transition. Yilmaz *et al.* [21] used the CSP method and achieved the maximum accuracy of 60.61% in case of Emotiv Epoc headset and 86.5% for wet gel electrodes. Athanasiou *et al.* [12] showed that the real and imaginary similarity for hand motion and foot motion is 85.71% and 71.41%, respectively, while the hand and foot motion’s discrimination for real is 28.57% and 14.28%, respectively. However, there are few studies about the MI applied on the patient with skeletal pain. Comparing with the previous work for classification between MI and ME [72], our results achieved a better accuracy (88%). In addition, the number of subjects were ten (healthy participants), while 71 patients participated in our study. Furthermore, the motions include three simple right upper limb movement, while our motions include left and right trunk bending.

In our experiment, we used the Emotiv EPOC equipment. Martinez-Leon *et al.* [74] provided an assessment of the Emotiv EPOC on the MI problem and showed that the performance of this headset was comparable to that found in professional devices when using the same number of sensors and sensor positions for a three status MI cognitive process.

The system accuracy was very similar for the Emotiv EPOC datasets whereas quite different for BCI Competition datasets (ranging from 59.45% to 83.19%). Schiatti *et al.* [75] used Emotiv EPOC for MI identification, and presented that limiting the analysis to EPOC channels caused a decrease of classification accuracy. The best classification accuracies were 62%, 61.5% and 63% respectively for three subjects. However, in our study, we used deep learning method and achieved a better performance. Compared to these works, our results using the deep learning method CNN and BiLSTM achieved comparable results considering the Emotiv equipment’s data.

The previous fMRI test used a few electrodes (C3, C4, Cz) to capture the corresponding EEG patterns for MI [76]. Other use 7 electrodes (CP1, CPz, CP2, C1, Cz, C2, FC1, FCz, and FC2) to focus on [12]. Although the Emotiv EPOC doesn’t have any electrodes placed on the supposed optimal spot for MI BCI, Emotiv EPOC is still can be used for MI BCI [77]. There are a few studies about the Emotiv EPOC on the MI. For instance, Osama and Aslam [78] used FC5 electrode’s signal as feedback. E. Fatmawati *et al.* [79] extracted features of alpha-frequency, beta frequency, mu maximum power and maximum beta power using probabilistic neural network (PNN) on F3, F4, FC5, FC6 electrode components, and testing accuracy achieved 82.6%–87.6%. Stock and Balbinot [80] used FC5, FC6, P7 and P8 of the 10–20 system, and a discussion about the differences of using C3, C4, P3, and P4 position is proposed. The maximum classification results for the proposed experiment and for the BCI Competition dataset were, respectively, 79% and 85%. In our experiment, FC5, FC6, AF3, AF4 electrode showed significant difference due to the massage therapy.

Interestingly, the SampEn of delta, alpha and beta rhythm all decreased in real motion compared to that in MI in four conditions. It indicated that the complexity of ME was more than that of MI in delta, alpha and beta rhythms, and less in theta rhythm. Compared with the SampEn and CSP feature, BiLSTM method received the highest classification accuracy (0.89) for the MI with trunk left/right bending before massage.

Study evidence showed that patients with lesions in the parietal and frontal cortices have difficulty performing MI, though they had ability to perform MI despite chronic or severe motor impairments [16]. In our study, for patients with chronic skeletal pain, they have mild to moderate level of dysfunction. Further studies are need to be investigated in the field.

## V. Conclusion

In this study, we investigated the effectiveness of massage on the four rhythms for MI and ME, and its classification between the left bending and right bending. We conducted an experiment on 71 patients with skeletal muscle pain, using MI and ME tasks before and after massage. The averaged SampEn values of four rhythms decreased in almost fourteen channels for five statuses (quiet, MI with left/right bending, ME with left/right bending). It indicated that massage alleviated the pain for the patients of skeletal pain after massage. The SampEn values of the delta, alpha and beta rhythm were higher in ME than in MI with the same motion, while SampEn values of the theta rhythm were smaller in ME than in MI with the same motion. PDI scores showed significant difference in alpha and beta rhythms before and after massage in different motions (MI and ME). The deep learning method achieved comparable accuracy to existing methods in the literature. It showed the effectiveness of massage not only in the quiet status, but also in the MI and ME.
